# ProbPFP: a multiple sequence alignment algorithm combining hidden Markov model optimized by particle swarm optimization with partition function

**DOI:** 10.1186/s12859-019-3132-7

**Published:** 2019-11-25

**Authors:** Qing Zhan, Nan Wang, Shuilin Jin, Renjie Tan, Qinghua Jiang, Yadong Wang

**Affiliations:** 1 School of Computer Science and Technology, Harbin Institute of Technology, Harbin, 150001 China; 2 Department of Mathematics, Harbin Institute of Technology, Harbin, 150001 China; 3 School of Life Science and Technology, Harbin Institute of Technology, Harbin, 150001 China

**Keywords:** Particle swarm optimization, Hidden Markov Model, Partition function, Multiple sequence alignment

## Abstract

**Background:**

During procedures for conducting multiple sequence alignment, that is so essential to use the substitution score of pairwise alignment. To compute adaptive scores for alignment, researchers usually use Hidden Markov Model or probabilistic consistency methods such as partition function. Recent studies show that optimizing the parameters for hidden Markov model, as well as integrating hidden Markov model with partition function can raise the accuracy of alignment. The combination of partition function and optimized HMM, which could further improve the alignment’s accuracy, however, was ignored by these researches.

**Results:**

A novel algorithm for MSA called ProbPFP is presented in this paper. It intergrate optimized HMM by particle swarm with partition function. The algorithm of PSO was applied to optimize HMM’s parameters. After that, the posterior probability obtained by the HMM was combined with the one obtained by partition function, and thus to calculate an integrated substitution score for alignment. In order to evaluate the effectiveness of ProbPFP, we compared it with 13 outstanding or classic MSA methods. The results demonstrate that the alignments obtained by ProbPFP got the maximum mean TC scores and mean SP scores on these two benchmark datasets: SABmark and OXBench, and it got the second highest mean TC scores and mean SP scores on the benchmark dataset BAliBASE. ProbPFP is also compared with 4 other outstanding methods, by reconstructing the phylogenetic trees for six protein families extracted from the database TreeFam, based on the alignments obtained by these 5 methods. The result indicates that the reference trees are closer to the phylogenetic trees reconstructed from the alignments obtained by ProbPFP than the other methods.

**Conclusions:**

We propose a new multiple sequence alignment method combining optimized HMM and partition function in this paper. The performance validates this method could make a great improvement of the alignment’s accuracy.

## Background

In bioinformatics, multiple sequence alignment is a foundermental conception. It aim to align more than two biomolecular sequences and applied for various biological analysis tasks, for example, protein structure prediction and phylogenetic inference [1]. Using MSA to find sequence differences can assist in the construction and annotation of biological ontologies, for example, the largest ontology in the world, Gene Ontology [2], on which researchers conduct a lot of works [3–7]. For the purpose of extracting and sharing knowledge of alignment, researchers established some ontologies based on multiple sequence alignment [8]. In addition, multiple sequence alignment could help to call SNP and thus to find disease-related gene variants [9–13].

There are many types of methods for multiple sequence alignment, and most of them are progressive [1]. Using a progressive method to align a set of sequences, first of all, for each paired sequence, we need to do pairwise alignment, then to compute the distance of the pair. A distance matrix was constituted from the distances of every pair. Subsequently, a guide tree was generated on the basis of the distance matrix. As the last step, on the ground of the provided order, which offered by the guide tree, profile-profile alignment was executed progressively.

For two sequences, the pairwise alignment task simply applies dynamic programming. And the scoring function for dynamic programming is usually based on a substitution matrix, for example, BLOSUM62 and PAM250 for protein sequences. In the multiple sequence alignment problems, when we need to align given sequences *x* and *y*, also the algorithms apply dynamic program, however the scoring function is not simply based on certain substitution matrix any more, since if residue *x*_*i*_ should be aligned with residue *y*_*j*_ is not just concerned about sequences *x* and *y* but also concerned about others. Numerous algorithms utilize the posterior probability *P*(*x*_*i*_∼*y*_*j*_|*x*,*y*) to compute the substitution scores. *P*(*x*_*i*_∼*y*_*j*_|*x*,*y*) represent the probability that residue on position *x*_*i*_ in sequence *x* and residue on position *y*_*j*_ in sequence *y* are matched in the “true” multiple sequence alignment [14].

For the sake of calculating the posterior probability, a large number of approaches are practiced by different algorithms. Among those considerable amount of progressive alignment algorithms, most of them apply Hidden Markov Model to calculate the posterior probability, for example, ProbCons [15]. But in the meantime, some algorithms apply other probability consistency approaches, for instance, partition function, which was applied by Probalign [16] to calculate the posterior probability.

Howell et al. [17] and McCaskill et al. [18] use partition function to predict RNA secondary structure. Song et al. [19] use partition function to align RNA pseudoknot structures. Using partition function to do alignment was pioneered by Miyazawa [20]. Wolfsheimer et al. [21] studied the parameters partition function for the alignment. MSARC use a residue clustering method based on partition function to align multiple sequence [22]. Retzlaff et al. [23] use partition function as a part of calculation for partially local multi-way alignments. Partition function is a useful model for alignment.

Some algorithms apply integrated approaches, for instance, MSAProbs [24] and QuickProbs [25] calculate the posterior probability according to the combination of HMM and partition function, while for GLProbs [26], based on the mean of sequences’ identity in a set, the posterior probability was calculated adaptively. These papers indicated that, a preferable result will be produced by combining two or more types of posterior probability, while the one using a single type will produce worse result.

For the purpose of optimizing the parameters of HMM in MSA problem, many kinds of optimization algorithms are employed by various algorithms, such as Particle Swarm Optimization [27–30], Evolutionary Algorithms [31] and Simulated Annealing [32], to make the alignment’s accuracy improved.

Won et al. [33] use an evolutionary method to learn the HMM structure for prediction of protein secondary structure. Rasmussen et al. [27] use a particle swarm optimization—evolutionary algorithm hybrid method to train the hidden Markov model for multiple sequence alignment. Long et al. [28] and Sun et al. [29] use quantum-behaved particle swarm optimization method to train the HMM for MSA. And Sun et al. [30] also use an random drift particle swarm optimization methods to train the HMM for MSA.

Nevertheless, combination of the partition function and the optimized HMM was ignored by these studies. So, a novel algorithm for MSA called ProbPFP is presented in this paper. ProbPFP integrates the posterior probabilities yield by particle swarm optimized HMM and those yield by partition function.

We compared ProbPFP with 13 outstanding or classic approaches, that is, Probalign [16], ProbCons [15], DIALIGN [34], Clustal *Ω* [35], PicXAA [36], KALIGN2 [37], COBALT [38], CONTRAlign [39], Align-m [40], MUSCLE [41], MAFFT [42], T-Coffee [43], and ClustalW [44], according to the total column score and sum-of-pairs score. The results indicated that ProbPFP got the maximum mean scores among the two benchmark datasets SABmark [40] and OXBench [45], along with the second highest mean score on the dataset BAliBASE [46].

## Methods

### Maximal expected accuracy alignment and posterior probability

A lot of multiple alignment methods construct alignment with maximum expected accuracy. A dynamic program need to be executed to determine the expected accuracy. The substitution score for the dynamic programming is set as the posterior probability when two corresponding positions in each sequence are aligned. The posterior probability was denoted as *P*_*x*,*y*_(*x*_*i*_∼*y*_*j*_)=*P*(*x*_*i*_∼*y*_*j*_|*x*,*y*), then the dynamic programming will be executed according to the following formula.

1 $$ A(i,j)=\max \left\{\begin{array}{l} A(i-1,j-1)+P_{x,y}(x_{i}\sim y_{j}) \\ A(i-1,j) \\ A(i,j-1) \\ \end{array}\right.  $$

For two sequences *x* and *y*, the maximal expected accuracy alignment will be generated when the dynamic programming finished. The alignment will get a corresponding maximum global score *GS*(*x*,*y*)=*A*(|*x*|,|*y*|).

#### Posterior probability calculating by partition function

Partition function is a core concept in statistical physics. It is similar to path integral mathematically. By calculating the partition function, the microstates can be related to the macroscopic physical quantity. And all of the thermodynamic functions that characterize the equilibrium thermodynamic properties of the system can be represented by partition function.

In equilibrium, the distribution of particles at each energy level follows the Boltzmann distribution, as the formula below:

2 $$  P_{i}\propto e^{-\frac{\varepsilon_{i}}{kT}}  $$

*P*_*i*_ indicates the probability that the particle is at the *i*-th level, *T* represents the thermodynamic temperature of the particle system, *ε*_*i*_ represents the free energy of the *i*-th level, and *k* represents the Boltzmann constant.

According to the formula (2), *P*_*i*_ can be calculated by:

3 $$  P_{i}=\frac{e^{-{\varepsilon }_{i}/kT}}{\sum\limits_{j=1}^{M}{e^{-{\varepsilon}_{j}/kT}}}  $$

The denominator $Z=\sum \limits _{j=1}^{M}{e^{-{\varepsilon }_{j}/kT}}$ is the partition function, which is the weighted sum of microstates. It described how does the probability of various microstates distributed in the system, and the value of it characterizes the ratio of particles’ amount in the system to particles’ amount at the ground state.

The partition function used in probability theory, information theory and dynamical systems is the generalization of the definition of partition function in statistical mechanics.

For protein alignment, since “any scoring matrix essentially corresponds to a log-odds matrix” [47], the total score *A*(*l*) of an alignment *l* is proportional to the log-likelihood ratio of *l*. So, the probability of an alignment *l* is proportional to *e*^(*A*(*l*)/*T*)^ which is similar to the Boltzmann distribution [20], where *T* is a constant related to the original scoring matrix.

If *T* was treated as the thermodynamic temperature, and the total score of alignment as negative energy, the probability of an alignment *l* could be calculated by the partition function defined as below:

4 $$\begin{array}{*{20}l} Z&=\sum_{l\in L}e^{A(l)/T} \end{array} $$

5 $$\begin{array}{*{20}l} p(l)&=e^{A(l)/T}/Z \end{array} $$

while *L* represents the set of each possible alignment of sequence *x* and sequence *y*.

The partition function for partial sequences of *x*[1…*i*] and *y*[ 1…*j*] is denoted as *Z*_*i*,*j*_, and for that of *x*[*i*…|*x*|] and *y*[ *j*…|*y*|] as $Z_{i,j}^{\prime }$. Each one of them could use dynamic program to calculated from the beginning or the ending of the sequences. Then, the posterior probability of position *x*_*i*_ aligned to position *y*_*j*_ could be calculated by the formula as below:

6 $$ P_{x,y}(x_{i}\sim y_{j})=\frac{1}{Z}\,Z_{i-1,j-1}\,e^{s(x_{i},y_{j})/T}\,Z_{i+1,j+1}^{'}  $$

where *s*(*x*_*i*_,*y*_*j*_) represents the score of aligning residue *x*_*i*_ with residue *y*_*j*_, in the original scoring matrix.

#### Posterior probability calculating by pair-HMM

Pair-HMM was used by numerous multiple sequence alignment methods to calculate posterior probability. The posterior probability that the *i*-th residue in sequence *x* and the *j*-th residue in sequence *y* is aligned in the "true" alignment of *x* and *y* is defined by the formula below:

7 $$ \begin{aligned} P_{x,y}(x_{i}\sim y_{j})&=P(x_{i}\sim y_{j}\in l^{*}|x,y) \\ &=\sum_{l\in L}P(l|x,y)1\{x_{i}\sim y_{j}\in l\} \end{aligned}  $$

while *L* represents the set of each possible alignment of sequences *x* and *y*, *l*^∗^ represents the “true” alignment of them, and 1(*expr*) represents the indicator function which returns 1 if the *expr* is true or 0 if it is false.

The majority multiple sequence alignment methods on the basis of pair-HMM use the Forward and Backward algorithm to compute the posterior probability, as explained in [14].

Nevertheless, for estimating the model parameters of HMM, there are selected algorithms that use certain other optimization methods instead of utilizing the Forward and Backward algorithm, to prevent being trapped in local optima, for example, particle swarm optimization.

#### Posterior probability calculating by particle swarm optimized pair-HMM

Optimization algorithms are derived from computer science. Nowadays, they are extensively applied in various subjects, for example, life science and material science, and so on [48, 49]. Optimization algorithms, for example, particle swarm optimization and random walk [5, 50–52] are also widely used in bioinformatics.

PSO [53] is an optimization algorithm which is inspired by foraging behavior of a bird flock. For an optimization problem, a number of particles are set by PSO algorithm. Position and velocity are the basic properties of all particles. A particle’s position stand for a candidate solutions in the solution space of the problem. The velocity of a particle indicate where it will go next. The positions are assessed by a fitness function.

PSO algorithms move the particles to “better” positions iteratively, based on the best position that a particle have reached along with the best position that the whole swarm have reached.

In this approach, there exist a total of *n* particles. It possess a stochastically yielded position vector *x*_*i*_ and a stochastically yielded velocity vector *v*_*i*_ for each particle *i*. In the algorithm, the formula (8) was used to renew the velocity, and also formula (9) was used to renew the position:

8 $$\begin{array}{*{20}l} v_{i}^{k}&={wv}_{i}^{k}+f_{1}r_{1}\left(p_{i}^{k}-x_{i}^{k}\right)+f_{2}r_{2}\left(p_{g}^{k}-x_{i}^{k}\right)  \end{array} $$

9 $$\begin{array}{*{20}l} x_{i}^{k+1}&=x_{i}^{k}+v_{i}^{k}  \end{array} $$

In these formulas, *p*_*i*_ represents the best position that particle *i* achieved. *p*_*g*_ represents the global best position of the whole swarm achieved. *w* represents the inertia weight that dominates the affects of the previous velocity. *f*_1_ is the cognitive factor, while *f*_2_ is the social factor. *r*_1_ and *r*_2_ are variables that yielded randomly in [ 0,1].

The fitness of the global best position will be improved as the renewing procedure iteratively runs. The renewing procedure will be stopped when iterations reaches a previously given number or the fitness reaches a previously given value.

For hidden Markov model, if we consider the parameter set of it as the position in PSO, then it can be optimized by PSO. For HMM in MSA problem, once the parameters of HMM are computed, the posterior probabilities for MSA will be computed subsequently.

#### Posterior probability calculating by integrating different methods

In order to align two sequences by dynamic programming, the most important element is the substitution score. Numerous approaches are applied to compute the posterior probabilities, and thus to compute the substitution scores. Each approach has its own particular property and matches distinct aspect of alignments. To integrate more than one approach to calculate the posterior probability is a conventional practice. MSAProbs [24] integrate the partition function with HMM to calculate the posterior probabilities, while GLProbs [26, 54] calculate the posterior probabilities by integrating local, global and double affine pair-HMMs.

#### Posterior probability calculating by integrating particle swarm optimized pair-HMM and partition function

In this paper, a multiple sequence alignment method which is called ProbPFP is proposed, while the posterior probability is determined by integrating particle swarm optimized HMM and partition function.

PSO was applied by ProbPFP to optimize the gap open penalties, gap extend penalties and the initial distribution of MSA. Thus for HMM in ProbPFP, the initial probabilities was calculated based on the initial distribution, and the transition probabilities was calculated based on these two type of penalties.

As the first step, the parameters are yielded randomly following a uniform distribution. Subsequently, the hidden Markov model for MSA was constructed by applying these parameters and then was used to calculate the posterior probabilities. We applied these posterior probabilities as the substitution scores to execute pairwise alignment.

In this paper, the fitness function for PSO is defined as SoP, i.e.,the standard sum-of-pairs score, which is described as below:

10 $$ \begin{aligned} SoP&=\sum_{i=1}^{n}\sum_{j=i+1}^{n}Score(l_{i},l_{j}) \\ &=\sum_{i=1}^{n}\sum_{j=i+1}^{n}\sum_{k=1}^{|l|}s(r_{ik},r_{jk}) \end{aligned}  $$

In which, sequences *i* and *j* are aligned as *l*_*i*_ and *l*_*j*_ by inserting gaps to them. *r*_*ik*_ is a gap or a residue at the position *k* on aligned sequence *l*_*i*_. *s*(*r*_*ik*_,*r*_*jk*_) is the score for the two elements *r*_*ik*_ and *r*_*jk*_ at position *k*. If the two elements are all residues, it is the substitution score for this two types of residue. If one of the elements is gap, it is the penalty of gap open or extend. In this study, the substitution matrix is the commonly used BLOSUM62. The gap open penalty is set as -11, and the gap extend penalty as -1, since the two values for these penalties are extensively used.

In order to optimize the SoP score, we did a series of experiments to determine how many particles and how many iterations we need. We finally chose 10 particles for 30 iterations. The experiments are described in “results” section. After that, the final trained parameters are used to construct a hidden Markov model. We apply the model to compute the posterior probability and denote this type of posterior probability as $P_{x,y}^{a}(x_{i}\sim y_{j})$.

The posterior probability computed by the partition function are denoted as $P_{x,y}^{b}(x_{i}\sim y_{j})$, and the final posterior probability are defined as below:

11 $$ P_{x,y}(x_{i}\sim y_{j})=\sqrt{\frac{P_{x,y}^{a}(x_{i}\sim y_{j})^{2}+P_{x,y}^{b}(x_{i}\sim y_{j})^{2}}{2}}  $$

### Guide tree construction

Once the posterior probabilities were generated, they are used as substitution scores in dynamic programming method to align two corresponding sequences. We get a final global score for the two sequences through the dynamic programming. Using all of the scores, we establish a distance matrix from which we establish a guide tree to guide the subsequent alignment.

#### Distance matrix computation

Since in bioinformatics, similarity is an important concept, various approaches are developed to measure similarity on numerous research fields [55–60]. For alignment problems, the dynamic programming can be performed to generate the maximal expected accuracy alignment by applying Eq. 1 iteratively based on posterior probability. The corresponding maximal expected accuracy can be calculated as the following formula:

12 $$ GS(x,y)=A(|x|,|y|)  $$

It is the sum of posterior probabilities for every aligned residue pair on the yielded alignment of sequences *x* and *y*, so it indicates the similarity of this two sequences. And then, the distance of them can be defined as shown:

13 $$ dis(x,y)=1-GS(x,y)/min\{|x|,|y|\}  $$

The distance matrix of a set of sequences, was constituted by the distances for every pair of sequences.

#### Guide tree building from distance matrix

Guide tree is a binary tree, that each node has two children. Each leaf of guide tree stands for a sequence, each internal node stands for an alignment of the sequences that the leaves of the corresponding sub-tree represent, and the root represents the final alignment. It can be built according to the distance matrix by using various clustering methods, for example, UPGMA and Neighbor-Joining. We applied UPGMA, which is a greedy linear heuristic methods, to build the guide tree, in this study.

When the two closest remaining nodes *N*_*i*_ and *N*_*j*_ are united to a node *N*_*k*_, for any other node *N*_*l*_, the distance between *N*_*k*_ and *N*_*l*_ is defined as the average distance of each pair of sequences that one from *N*_*k*_ and another from *N*_*l*_.

14 $$ d_{kl}=\frac{\sum\limits_{x \in {N_{k}}} \sum\limits_{y \in {N_{l}}} d_{xy}}{|N_{k}| \cdot |N_{l}|}  $$

So it can be calculated by:

15 $$ d_{kl}=\frac{|N_{i}|d_{il}+|N_{j}|d_{jl}}{|N_{i}|+|N_{j}|}  $$

### Progressive alignment

Progressive alignment is the last procedure of ProbPFP. An unaligned sequence or the alignment of some aligned sequences is called profile. Starting from the set of original sequences, the core idea of progressive alignment is choosing the closest pair of profiles in the set and aligning them to generate a new profile to replace them in the set. As mentioned in the previous subsection, we learned that the aligning order is actually determined by the guide tree.

Before we apply progressive alignment, we first apply the probabilistic consistency transformation described in MSAProbs [24]. Probabilistic consistency transformation is a step to re-estimate the probabilities by considering the other sequences’ effect on the pairwise alignment. After that, as similar to pairwise alignment of two sequences, the profile-profile alignment also apply dynamic programming. It is intuitive that the substitution score for a pair of columns from these two profiles is determined by the mean of the posterior probability for every residue pair, that one residue located in the column from the first profile, while the other one located in the column from the second profile. The formula for the score is listed as below:

16 $$ Score(X_{i},Y_{j})=\frac{ \sum\limits_{x \in X,y \in Y}w_{x} w_{y} P^{'}(x_{i} \sim y_{j}|x,y) }{ \sum\limits_{x \in X,y \in Y}w_{x} w_{y} }  $$

where *X* and *Y* are profiles, *i* and *j* are the *i*-th and *j*-th columns. *P*^′^ is the transformed probabilistic matrix, and *w*_*x*_ and *w*_*y*_ are the weights which were calculated according to the methods in ClustalW [44].

We will execute the profile alignment progressively until there will be only one profile. The last profile will be the initial alignment that we seek for the set of sequences.

As the last step, we divide the alignment into two random groups and realignment them by profile alignment. After a fixed number of iterations (10 by default), we got the final alignment.

**Fig. 1:**
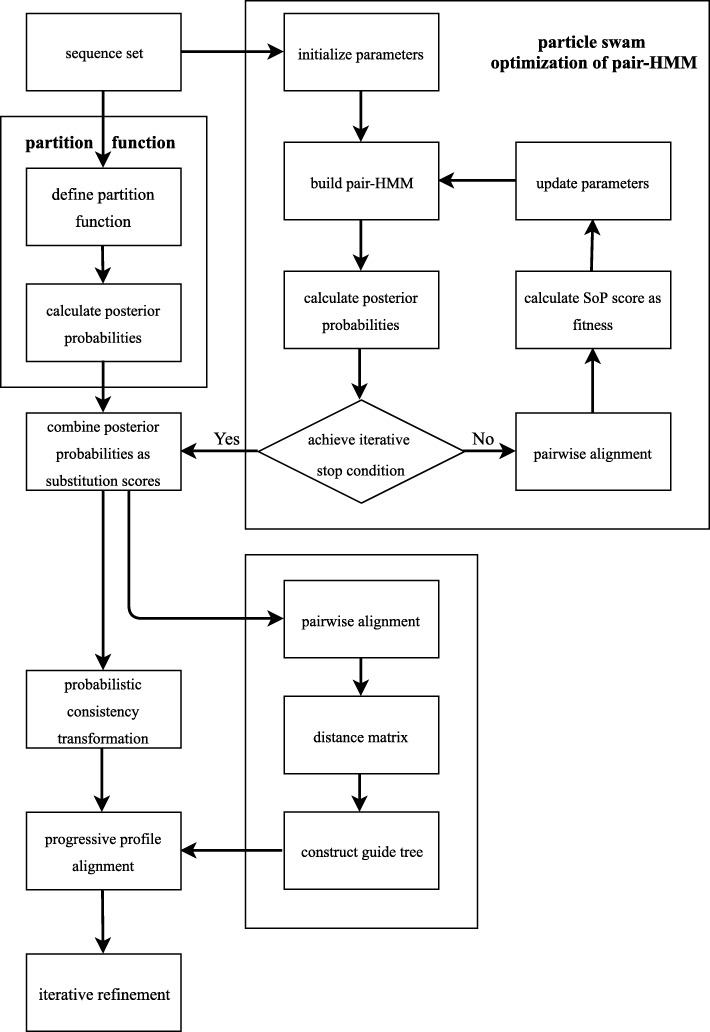
Framework of the ProbPFP algorithm

The steps for ProbPFP are displayed in Fig. 1.

## Results

We compared ProbPFP with 13 outstanding or classic MSA methods, i.e., Probalign, ProbCons, T-Coffee, PicXAA, CONTRAlign, COBALT, Clustal *Ω*, MUSCLE, KALIGN2, MAFFT, ClustalW, Align-m and DIALIGN. These 13 methods were all run with their default parameters. The particle swarm optimization in ProbPFP utilized 10 particles and iterated for 30 times.

**Fig. 2:**
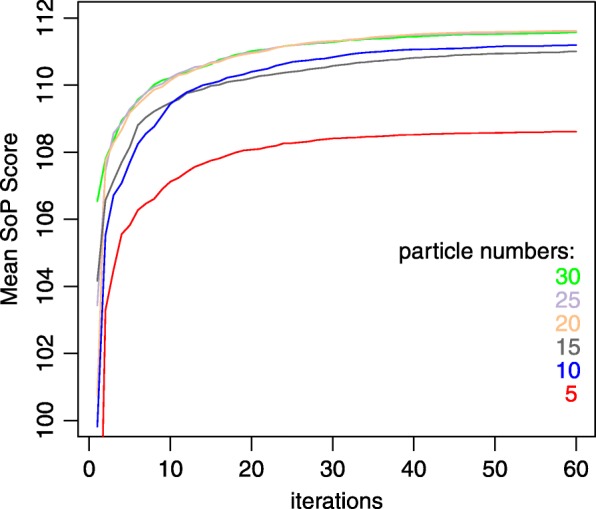
Comparison of mean SoP scores for different numbers of particles and iterations

The numbers of particles and iterations are determined by a series of experiments according to the SoP score on the RV11 and RV12 subsets of BAliBASE3 benchmark. To determine the number of particles and the number of iterations, we applied 5, 10, 15, 20, 25 and 30 particles to the families in RV11 and RV12, and iterated from 1 to 60 times. The mean SoP scores of this families are calculated. The results are described in Fig. 2. We noticed that the SoP scores increased a lot, as the number of particles increased from 5 to 10. But when the number increased from 10 to 15, 20, 25 or 30, the SoP scores increased only a little. In addition, when the number increased from 10 to 15, as the iterations increased, the SoP scores even decreased. So, we chose 10 particles which is enough.

From Fig. 2, we noticed that the SoP score increased as the number of iterations grow. But the increment speeds become slow after about 15 times, and even slower after about 30 times. So, we chose 30 iterations which is enough.

we applied these 14 algorithms to align the sequence sets in three commonly used protein multiple sequence alignment public benchmarks: SABmark 1.65, OXBench 1.3 and BAliBASE 3. These benchmarks were obtained from a collection which was downloaded from Robert C. Edgar’s personal website that is listed in the “Availability of data and materials” sections. Edgar gathered these benchmark datasets into the collection and converted the format of all these sequences to the convenient standard FASTA. In particular, only the RV11 subsets and the RV12 subsets in BAliBASE 3 and the Twilight Zone subsets and the Superfamily subsets in SABmark 1.65 were used in the comparison. As reported in [41], these subsets are consistent for experiments.

The algorithms were compared based on the total column score and sum-of-pairs score. For each benchmark and each algorithm, the mean of the TC scores of alignments for every family is calculated, as same as the mean of the SP scores.

Table 1 listed the mean TC scores and the mean SP scores on OXBench 1.3 of ProbPFP and the other 13 methods. The table demonstrated that ProbPFP got the maximum mean scores while Probalign got the second largest mean scores. Probalign calculated the posterior probabilities only by partition function model which “might be more successful in locating highly similar regions” [24], while ProbPFP do that by combining partition function with optimized HMM, and this strategy makes the score increased.

**Table 1:** Mean TC and SP Scores for 14 Aligners on OXBench

Aligner	Mean TC score	Mean SP score
ProbPFP	**81.70**	**90.15**
Probalign	*81.68*	*89.97*
ProbCons	80.86	89.68
T-Coffee	80.50	89.52
PicXAA	80.74	89.64
CONTRAlign	79.87	89.34
COBALT	79.73	88.96
Clustal *Ω*	79.99	88.91
MUSCLE	80.67	89.50
KALIGN	78.88	88.39
MAFFT	77.96	88.00
ClustalW	80.16	89.43
Align-m	76.06	86.95
DIALIGN	72.14	83.97

The scores in this table are multiplied by 100. In each column, the maximum score is highlighted in bold, while the second highest score is displayed between two asterisks

Table 2 listed the mean TC scores and the mean SP scores on BAliBASE 3. It indicated that ProbPFP got the second largest mean scores, and these scores were very close to the highest that Probalign got. “The partition function probabilistic model might be more successful in locating highly similar regions” [24] while “BAliBASE is heavily biased toward globally related protein families” [61]. We thought that is why Probalign got the highest scores. In this case, combining with optimized HMM might not benefit the scores but rather decrease them.

**Table 2:** Mean TC and SP Scores for 14 Aligners on BAliBASE

Aligner	Mean TC score	Mean SP score
ProbPFP	*67.03*	*82.50*
Probalign	**67.27**	**82.53**
ProbCons	65.22	81.55
T-Coffee	64.93	80.82
PicXAA	66.08	81.33
CONTRAlign	58.10	77.59
COBALT	57.49	76.08
Clustal *Ω*	59.38	75.96
MUSCLE	58.27	75.60
KALIGN	59.66	76.99
MAFFT	52.58	72.46
ClustalW	49.21	69.63
Align-m	56.04	71.45
DIALIGN	48.22	68.63

The scores in this table are multiplied by 100. In each column, the maximum score is highlighted in bold, while the second highest score is displayed between two asterisks

Table 3 listed the mean TC scores and the mean SP scores on SABmark. This table also indicated that ProbPFP got the maximum mean scores. Because most families in SABmark are divergent, Probalign didn’t get the second largest mean scores, but T-Coffee got the second largest mean TC score since it combined local and global alignment. The result shows that the combination strategy in our ProbPFP methods is also effective in divergent families.

**Table 3:** Mean TC and SP Scores for 14 Aligners on SABmark

Aligner	Mean TC score	Mean SP score
ProbPFP	**39.56**	**59.84**
Probalign	38.63	59.53
ProbCons	39.17	*59.69*
T-Coffee	*39.53*	59.14
PicXAA	39.11	59.37
CONTRAlign	35.59	57.54
COBALT	36.00	56.71
Clustal *Ω*	35.47	55.02
MUSCLE	33.47	54.51
KALIGN	33.22	52.13
MAFFT	32.57	52.63
ClustalW	31.37	51.92
Align-m	31.07	46.19
DIALIGN	27.11	47.09

The scores in this table are multiplied by 100. In each column, the maximum score is highlighted in bold, while the second highest score is displayed between two asterisks

Furthermore, we utilized ProbPFP to assist the rebuilding of the phylogenetic tree to assess the practicability of it. On 6 protein families extracted from the database TreeFam [62]. ProbPFP was compared with 4 other outstanding methods. The alignments that aligned by these 5 methods are passed to the analysis tool MEGA5 [63]. And in MEGA5, the phylogenetic trees of these 6 families are rebuilt by applying the maximum likelihood approach.

To assess the quality of the reconstructed phylogenetic trees, we need to calculate the distances between the reference trees with them. Here, we applied the commonly used partition metric (Robinson-Foulds metric). A better inferred tree has a smaller distance, since it is closer to the reference tree. Table 4 listed the Robinson-Foulds distances between the reference trees and the phylogenetic trees inferred from the alignments generated by this 5 aligners. It indicated that the trees computed from ProbPFP are with the smallest distances in 5 of the 6 tests.

**Table 4:** Robinson-Foulds Distances between the Inferred Phylogenetic Trees with the Reference Tree

TreeFam ID	ProbPFP	MUSCLE	MSAProbs	Clustal **Ω**	T-Coffee
TF101116 (104)	**0.87**	**0.87**	0.97	0.98	0.90
TF105063 (133)	**0.80**	0.83	0.85	0.84	0.84
TF105629 (88)	**0.62**	0.66	0.67	0.68	0.65
TF105895 (89)	**0.48**	0.53	0.53	0.56	0.51
TF106377 (26)	**0.39**	0.48	0.48	0.48	0.43
TF101222 (48)	0.71	**0.67**	**0.67**	0.78	0.76

For each family, the number in the parentheses after the ID represents the sequences amount of the family. The smallest distances are highlighted in bold, in each row

## Discussion

ProbPFP was compared with 13 outstanding or classic MSA methods based on TC and SP Scores. It achieved the highest mean TC and SP Scores among these 14 methods on the benchmark Sabre and OXBench. And on dataset BAliBASE, ProbPFP achieved the second highest mean TC and SP Scores and are very close to the highest scores that Probalign obtained.

To illustrate the practicability of ProbPFP, We also compared ProbPFP with 4 leading aligners according to phylogenetic tree reconstruction. Among the 6 tests, there are 5 tests in which the trees constructed from alignments yielded by ProbPFP are nearest to those reference trees.

It can be seen that combining PSO optimized HMM with partition function could make a great improvement of the alignment quality.

## Conclusions

The accuracy of sequence alignment could be raised by optimizing the parameters of HMM for multiple sequence alignment. It could also be improved by combining hidden Markov model with partition function. In this paper, we propose a new MSA method, ProbPFP, that integrates the HMM optimized by PSO with the partition function. The performance validates this method could make a great improvement of the alignment’s accuracy.

## Data Availability

The public datasets of MSA benchmarks used during the current study are available from http://www.drive5.com/bench.

## Abbreviations

HMM: Hidden Markov model
MEGA5: Molecular evolutionary genetics analysis tool 5
MSA: Multiple sequence alignment
PSO: Particle swarm optimization
RF: Robinson-foulds
SP: Sum-of-pairs
TC: Total column
UPGMA: Unweighted pair-group method with arithmetic means
